# Supplementary material for: Effects of water quality, sanitation, handwashing and nutritional interventions on enteric pathogen seroconversion in rural Bangladesh: a cluster randomised trial
Source: medRxiv. 2026 Nov 1:2026.10.30.26366885. Preprint. [Version 1] doi: 10.64898/2026.10.30.26366885 (PMC13734212; doi:10.64898/2026.10.30.26366885)
Supplement: Supplementary file 1 [file media-1.pdf]

Supplementary material

Effects of water quality, sanitation, handwashing and nutritional interventions on enteric pathogen seroconversion in rural Bangladesh: a cluster randomized trial

Mahbubur Rahman*, Pearl Anne Ante-Testard*, Richelle W. Kihoro, Henry Kanyi, Henry Emisiko, Huiyu Hu, Everlyn Kamau, E. Brook Goodhew, Chabier Coleman, Shahjahan Ali, Md Ziaur Rahman, Abul Kasham Shoab, Md. Rana Miah, Sania Ashraf, Sarker M Parvez, Md. Mostofa Shakil, Samantha J. Bents, Julianna Colado, Nikolina Walas, Jessica A. Grembi, Richelle C. Charles, Edward T. Ryan, Claire Munroe, Robert L. Atmar, Jade Benjamin-Chung, Ayse Ercumen, Andrew N. Mertens, Audrie Lin, Patrick J. Lammie, Christine P. Stewart, John M. Colford Jr., Stephen P. Luby, Sammy M. Njenga, Rashidul Haque, Jeffrey W. Priest, Benjamin F. Arnold (*equal contribution)

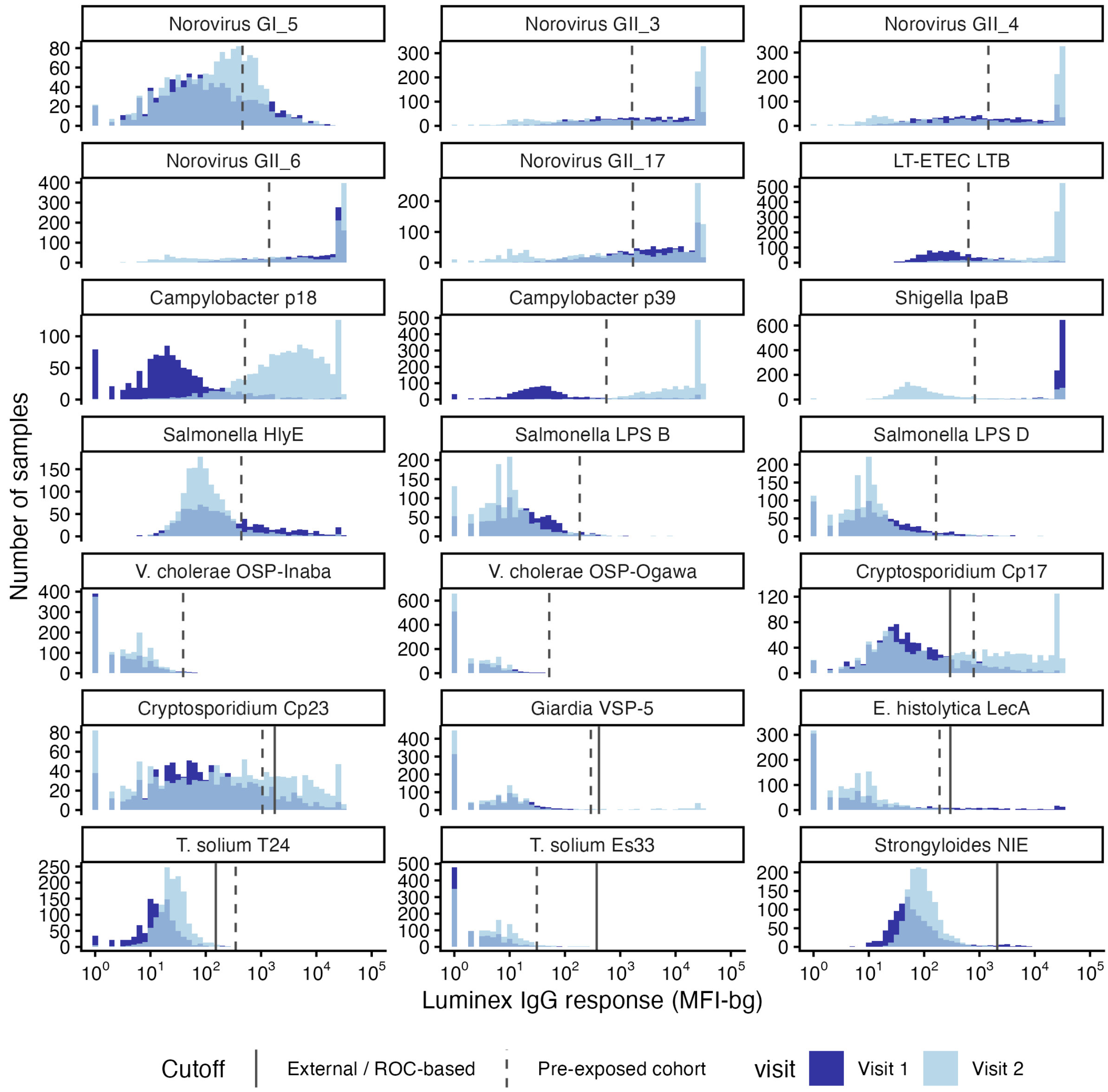

Supplementary Figure 1. Luminex IgG response (MFI-bg) to enteric pathogens during visits 1 and 2 in the WASH Benefits Bangladesh environmental enteric dysfunction substudy. Children were median ages 3 months (visit 1) and 14 months (visit 2) at the time of measurement. Vertical lines mark seropositivity cutoffs derived using external samples (solid) for protozoan and helminth pathogens, and from longitudinal boosting in a pre-exposed cohort approach (dashed).

Supplementary Table 1: Baseline characteristics by intervention groups.

	Control (N=897)	WASH (N=534)	Nutrition (N=495)	WASH + Nutrition (N=527)
**Maternal age (years)** Median [Min, Max]	23 [15, 43]	25 [15, 44]	23 [15, 45]	23 [14, 43]
**Maternal education** No education	119 (13.3%)	70 (13.1%)	81 (16.5%)	82 (15.6%)
Primary (1-5y)	280 (31.4%)	172 (32.2%)	145 (29.5%)	167 (31.7%)
Secondary (>5y)	493 (55.3%)	292 (54.7%)	266 (54.1%)	278 (52.8%)
**Maternal height (cm)** Median [Min, Max]	151 [134, 167]	150 [136, 164]	150 [127, 168]	150 [133, 167]
**Paternal education** No education	252 (28.3%)	164 (30.9%)	150 (30.5%)	157 (29.8%)
Primary (1-5y)	265 (29.8%)	152 (28.6%)	155 (31.6%)	170 (32.3%)
Secondary (>5y)	373 (41.9%)	215 (40.5%)	186 (37.9%)	199 (37.8%)
**Works in agriculture**	273 (30.6%)	160 (30.0%)	167 (33.9%)	162 (30.8%)
**International wealth index** Median [Min, Max]	33.7 [0.00, 63.0]	31.7 [4.66, 63.0]	29.6 [0, 63.0]	31.5 [4.66, 63.0]
**Number of households per compound** Mean (SD)	11.0 (6.17)	11.1 (5.90)	11.6 (6.51)	11.0 (6.48)
**Number of people per compound** Mean (SD)	4.73 (2.23)	4.62 (2.06)	4.65 (2.10)	4.76 (2.12)
**Primary drinking water source (tubewell)**	691 (77.5%)	424 (79.4%)	367 (74.6%)	391 (74.2%)
**Distance to primary water source (min)** Mean (SD)	1.29 (20.1)	0.979 (5.37)	0.616 (1.29)	0.706 (1.99)
**Reported storing drinking water**	404 (45.3%)	222 (41.6%)	209 (42.5%)	253 (48.0%)
**Reported treating water today or yesterday**	3 (0.3%)	0 (0%)	0 (0%)	2 (0.4%)
**Latrine** No latrine	408 (48.6%)	245 (48.1%)	219 (47.1%)	245 (49.6%)
Latrine without water seal	267 (31.8%)	184 (36.1%)	161 (34.6%)	174 (35.2%)
Latrine with water seal	164 (19.5%)	80 (15.7%)	85 (18.3%)	75 (15.2%)
**Primary handwashing location has water and soap**	190 (22.9%)	119 (24.2%)	99 (21.9%)	121 (24.6%)
**HFIAS food insecurity** Moderate/severe	201 (22.5%)	118 (22.1%)	104 (21.1%)	105 (19.9%)

WASH=combined water, sanitation and handwashing; SD=standard deviation; HFIAS=Household Food Insecurity Access Scale.

Supplementary Table 2: Measurement of intervention adherence by study group at enrollment, year 1 and year 2 follow-up.

Indicator	Round	WASH	No WASH	Nutrition	No Nutrition
Number of compounds assessed	Enrollment	1388/1388 (100%)	2081/2081 (100%)	1385/1385 (100%)	2084/2084 (100%)
	Year 1	1205/1388 (87%)	1732/2081 (83%)	1181/1385 (85%)	1756/2084 (84%)
	Year 2	1174/1388 (85%)	1712/2081 (82%)	1160/1385 (84%)	1726/2084 (83%)
Stored drinking water	Enrollment	635/1388 (46%)	967/2081 (46%)	632/1385 (46%)	970/2084 (47%)
	Year 1	1165/1205 (97%)	732/1732 (42%)	806/1181 (68%)	1091/1756 (62%)
	Year 2	1127/1174 (96%)	710/1712 (41%)	794/1160 (68%)	1043/1726 (60%)
Stored drinking water has detectable free chlorine (>0.1 mg/L)	Enrollment	..	..	..	..
	Year 1	939/1180 (80%)	..	..	..
	Year 2	972/1157 (84%)	..	..	..
Latrine with a functional water seal	Enrollment	307/1155 (27%)	541/1758 (31%)	338/1160 (29%)	510/1753 (29%)
	Year 1	1137/1202 (95%)	457/1579 (29%)	713/1130 (63%)	881/1651 (53%)
	Year 2	1128/1170 (96%)	487/1579 (31%)	724/1109 (65%)	891/1640 (54%)
No visible feces on latrine slab or floor	Enrollment	587/1296 (45%)	956/1956 (49%)	629/1292 (49%)	914/1960 (47%)
	Year 1	1049/1203 (87%)	991/1643 (60%)	860/1153 (75%)	1180/1693 (70%)
	Year 2	979/1170 (84%)	925/1626 (57%)	808/1124 (72%)	1096/1672 (66%)
Handwashing location has soap	Enrollment	304/1286 (24%)	446/1900 (23%)	301/1284 (23%)	449/1902 (24%)
	Year 1	1082/1204 (90%)	455/1515 (30%)	708/1111 (64%)	829/1608 (52%)
	Year 2	1071/1174 (91%)	515/1712 (30%)	735/1160 (63%)	851/1726 (49%)
Lipid Nutrient Supplement sachets consumed (% expected)*	Enrollment	..	..	..	..
	Year 1	94%	93%	94%	..
	Year 2	93%	94%	93%	..

Data are n (%) or %.
'..' = not measured or not interpretable on this margin.
Free chlorine reported for the WASH group only; *LNS % are conditional on being in a Nutrition cluster. Both not measured at enrolment.

Supplemental Table 3. Enteric pathogen seroprevalence at the 24-month visit by individual treatment group and main-effect comparison.

Pathogen	Intervention group	Seroprevalence n/N (%)	Main effect group	Seroprevalence n/N (%)	RD (95% CI)	p-value
**Viruses**						
***Norovirus* GI/GII**	Control	863/897 (96.2%)	No WASH	1336/1391 (96%)	Ref	—
	WASH	515/534 (96.4%)	WASH	1029/1061 (97%)	0.9 (-0.3, 2.1)	0.229
	Nutrition	473/494 (95.7%)	No Nutrition	1378/1431 (96.3%)	Ref	—
	WASH + Nutrition	514/527 (97.5%)	Nutrition	987/1021 (96.7%)	0.3 (-0.9, 1.5)	0.700
**Bacteria**						
**LT-ETEC (LTB)**	Control	891/896 (99.4%)	No WASH	1382/1391 (99.4%)	Ref	—
	WASH	531/534 (99.4%)	WASH	1053/1061 (99.2%)	-0.0 (-0.6, 0.5)	0.936
	Nutrition	491/495 (99.2%)	No Nutrition	1422/1430 (99.4%)	Ref	—
	WASH + Nutrition	522/527 (99.1%)	Nutrition	1013/1022 (99.1%)	-0.3 (-0.8, 0.3)	0.375
***Campylobacter* (p18,p39)**	Control	827/896 (92.3%)	No WASH	1292/1391 (92.9%)	Ref	—
	WASH	499/534 (93.4%)	WASH	996/1061 (93.9%)	0.8 (-1.0, 2.6)	0.467
	Nutrition	465/495 (93.9%)	No Nutrition	1326/1430 (92.7%)	Ref	—
	WASH + Nutrition	497/527 (94.3%)	Nutrition	962/1022 (94.1%)	1.2 (-0.6, 3.0)	0.287
***Shigella*/EIEC (IpaB)**	Control	719/896 (80.2%)	No WASH	1099/1391 (79%)	Ref	—
	WASH	397/534 (74.3%)	WASH	790/1061 (74.5%)	-4.5 (-7.6, -1.5)	0.011
	Nutrition	380/495 (76.8%)	No Nutrition	1116/1430 (78%)	Ref	—
	WASH + Nutrition	393/527 (74.6%)	Nutrition	773/1022 (75.6%)	-1.5 (-4.5, 1.6)	0.402
***S. enterica* typhi (HlyE)**	Control	107/896 (11.9%)	No WASH	176/1391 (12.7%)	Ref	—
	WASH	83/534 (15.5%)	WASH	156/1061 (14.7%)	2.1 (-0.5, 4.6)	0.177
	Nutrition	69/495 (13.9%)	No Nutrition	190/1430 (13.3%)	Ref	—
	WASH + Nutrition	73/527 (13.9%)	Nutrition	142/1022 (13.9%)	0.2 (-2.4, 2.9)	0.888
***S. enterica* (LPS B,D)**	Control	136/896 (15.2%)	No WASH	211/1390 (15.2%)	Ref	—
	WASH	66/534 (12.4%)	WASH	118/1061 (11.1%)	-4.0 (-6.5, -1.5)	0.008
	Nutrition	75/494 (15.2%)	No Nutrition	202/1430 (14.1%)	Ref	—
	WASH + Nutrition	52/527 (9.9%)	Nutrition	127/1021 (12.4%)	-1.3 (-3.9, 1.3)	0.389
***V. cholerae* (OSP-Inaba/Ogawa)**	Control	29/896 (3.2%)	No WASH	46/1390 (3.3%)	Ref	—
	WASH	15/534 (2.8%)	WASH	27/1061 (2.5%)	-0.8 (-2.2, 0.5)	0.292
	Nutrition	17/494 (3.4%)	No Nutrition	44/1430 (3.1%)	Ref	—
	WASH + Nutrition	12/527 (2.3%)	Nutrition	29/1021 (2.8%)	-0.2 (-1.6, 1.1)	0.752

**Protozoa**						
***Cryptosporidium* (Cp17,Cp23)**	Control	562/896 (62.7%)	No WASH	892/1391 (64.1%)	Ref	—
	WASH	319/534 (59.7%)	WASH	660/1061 (62.2%)	-2.9 (-6.5, 0.7)	0.173
	Nutrition	330/495 (66.7%)	No Nutrition	881/1430 (61.6%)	Ref	—
	WASH + Nutrition	341/527 (64.7%)	Nutrition	671/1022 (65.7%)	4.1 (0.4, 7.8)	0.051
***Giardia* (VSP-5)**	Control	286/897 (31.9%)	No WASH	443/1391 (31.8%)	Ref	—
	WASH	153/534 (28.7%)	WASH	300/1061 (28.3%)	-3.4 (-6.7, -0.2)	0.069
	Nutrition	157/494 (31.8%)	No Nutrition	439/1431 (30.7%)	Ref	—
	WASH + Nutrition	147/527 (27.9%)	Nutrition	304/1021 (29.8%)	-0.4 (-3.7, 2.8)	0.820
***E. histolytica* (LecA)**	Control	13/896 (1.5%)	No WASH	24/1391 (1.7%)	Ref	—
	WASH	7/534 (1.3%)	WASH	22/1061 (2.1%)	0.2 (-0.7, 1.2)	0.703
	Nutrition	11/495 (2.2%)	No Nutrition	20/1430 (1.4%)	Ref	—
	WASH + Nutrition	15/527 (2.8%)	Nutrition	26/1022 (2.5%)	1.0 (0.1, 2.0)	0.052
**Helminths**						
***T. solium* (T24,Es33)**	Control	19/896 (2.1%)	No WASH	36/1390 (2.6%)	Ref	—
	WASH	15/534 (2.8%)	WASH	32/1061 (3%)	0.2 (-1.0, 1.4)	0.739
	Nutrition	17/494 (3.4%)	No Nutrition	34/1430 (2.4%)	Ref	—
	WASH + Nutrition	17/527 (3.2%)	Nutrition	34/1021 (3.3%)	0.9 (-0.3, 2.1)	0.195
***S. stercoralis* (NIE)**	Control	1/896 (0.1%)	No WASH	2/1391 (0.1%)	Ref	—
	WASH	1/534 (0.2%)	WASH	3/1061 (0.3%)	0.1 (-0.2, 0.4)	0.616
	Nutrition	1/495 (0.2%)	No Nutrition	2/1430 (0.1%)	Ref	—
	WASH + Nutrition	2/527 (0.4%)	Nutrition	3/1022 (0.3%)	0.1 (-0.2, 0.4)	0.633

Note:
n/N (%) shows observed seropositive counts and percentages.
Risk differences are marginal main-effect estimates reported in percentage points.
P values are two-sided permutation p-values from the primary main-effect models.

Pathogen	Nutrition	No Nutrition	Diff (95% CI)	p-value	FDR p-value
Norovirus GI/GII	96.7%	96.4%	0.3 (-0.9, 1.5)	0.70	0.89
LT-ETEC (LTB)	99.2%	99.4%	-0.3 (-0.8, 0.3)	0.38	0.69
Campylobacter (p18,p39)	94.0%	92.8%	1.2 (-0.6, 3.0)	0.29	0.69
Shigella/EIEC (IpaB)	75.9%	77.4%	-1.5 (-4.5, 1.6)	0.40	0.69
S. enterica typhi (HlyE)	14.1%	13.9%	0.2 (-2.4, 2.9)	0.89	0.89
S. enterica (LPS B,D)	12.5%	13.9%	-1.3 (-3.9, 1.3)	0.39	0.69
V. cholerae (OSP-Inaba/Ogawa)	2.8%	3.1%	-0.2 (-1.6, 1.1)	0.75	0.89
Cryptosporidium (Cp17,Cp23)	65.4%	61.2%	4.1 (0.4, 7.8)	0.05	0.31
Giardia (VSP-5)	30.0%	30.5%	-0.4 (-3.7, 2.8)	0.82	0.89
E. histolytica (LecA)	2.4%	1.4%	1.0 (0.1, 2.0)	0.05	0.31
T. solium (T24,Es33)	3.3%	2.4%	0.9 (-0.3, 2.1)	0.20	0.69

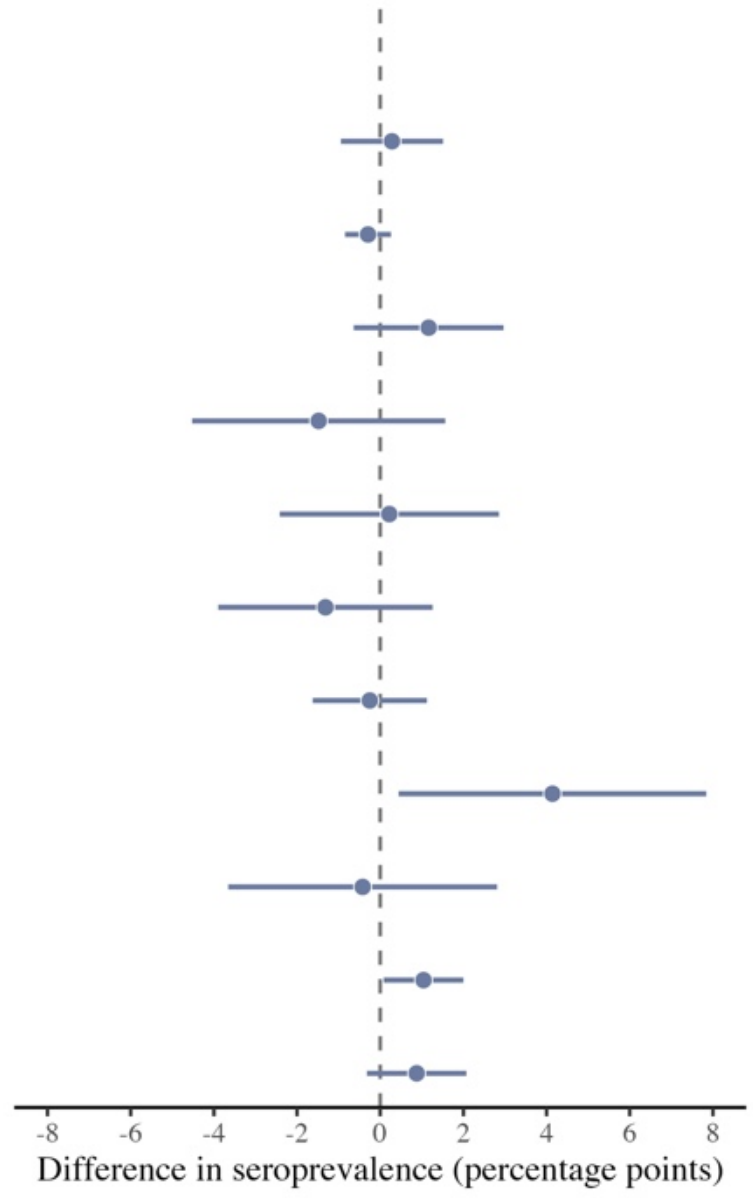

Supplementary Figure 2. Nutrition intervention effects on enteric pathogen seroprevalence at median age 24 months. Effects estimated as marginal means and treatment effects for clusters that received Nutrition (Nutrition, WASH + Nutrition) compared with clusters that did not (WASH, Control)**.** Two-sided permutation p-values were estimated by permuting treatment assignments (10,006 replicates). P-values were adjusted for multiple comparisons using the Benjamini-Hochberg procedure across the 12 comparisons (5% false discovery rate). Excluded results for *S. stercoralis (NIE)* because seroprevalence was low (<1%).

WASH=combined water, sanitation and handwashing; Diff=difference; 95% CI=95% confidence interval; FDR=False Discovery Rate.

A

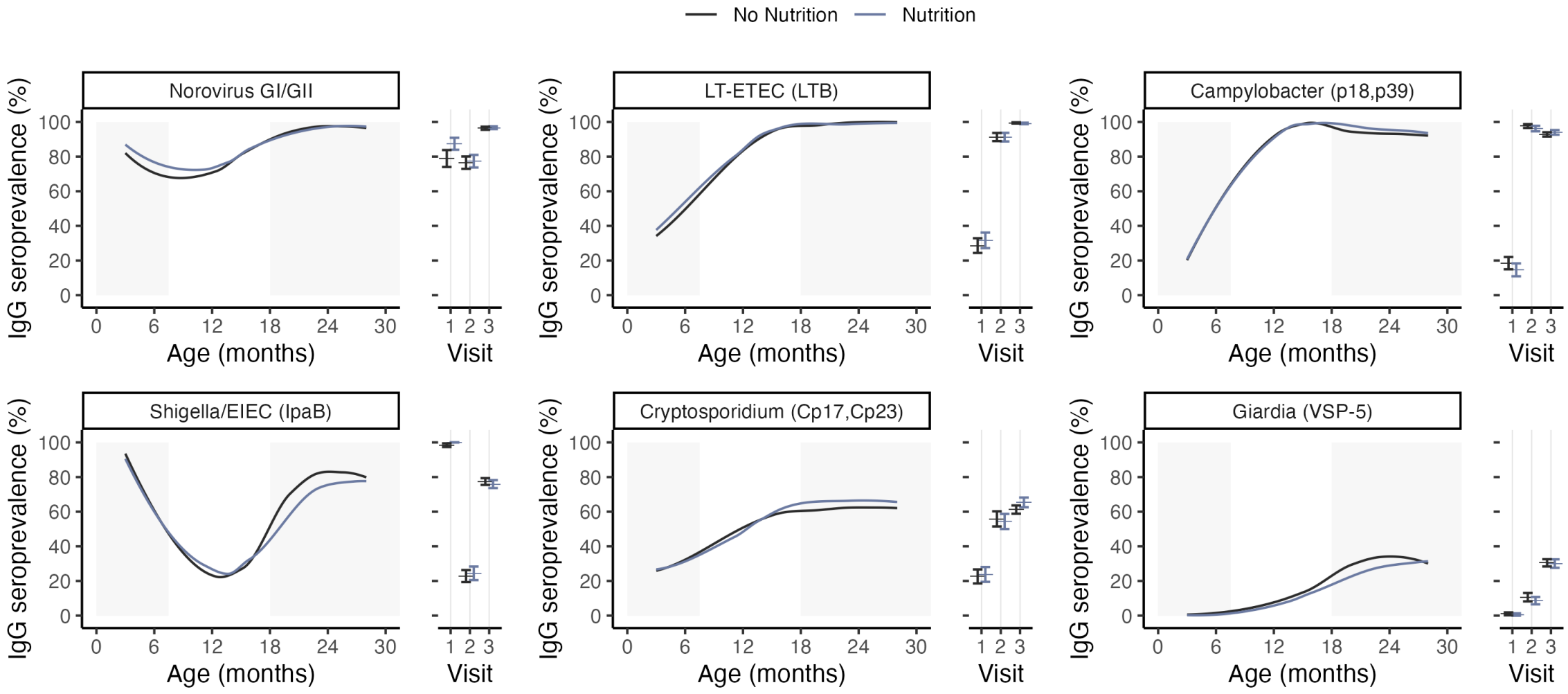

B

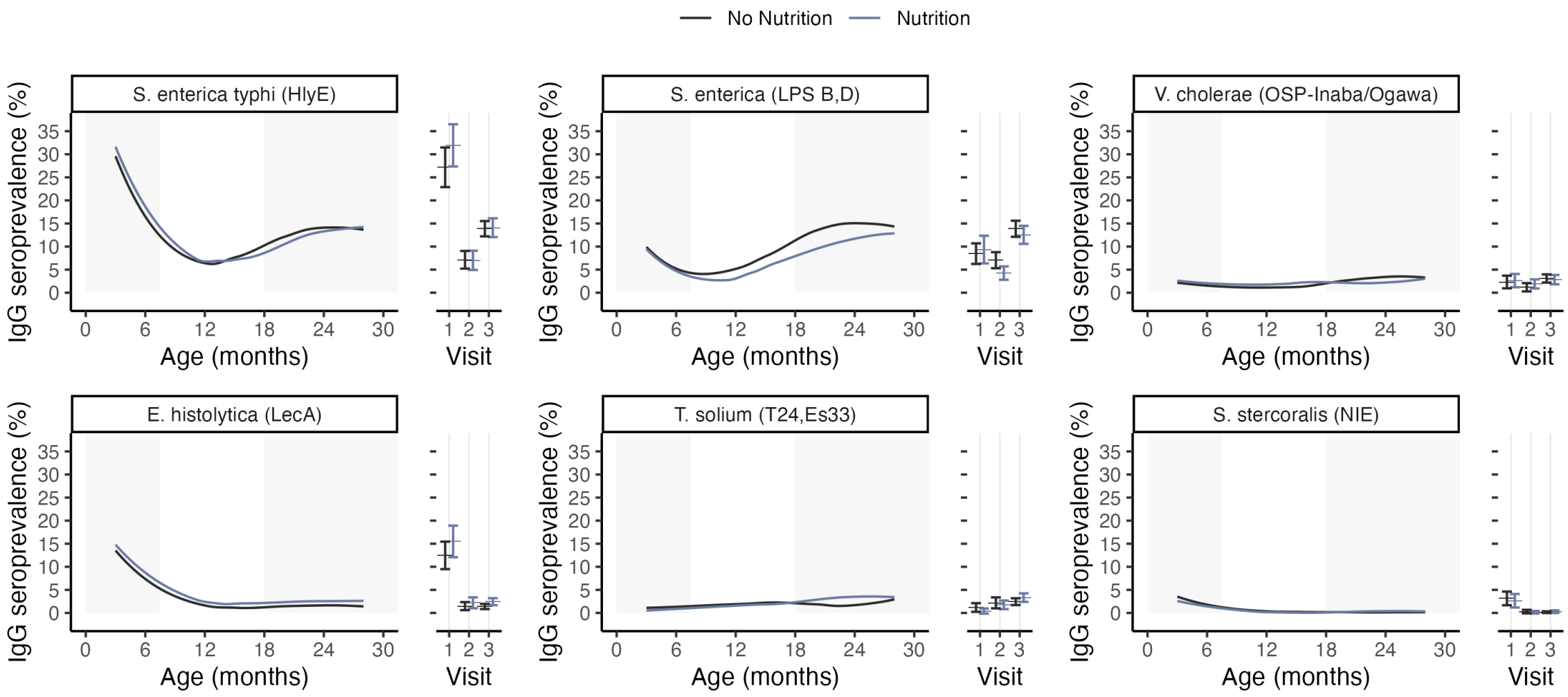

Supplementary Figure 3. Enteric pathogen seroprevalence by age and Nutrition intervention group, Bangladesh 2013-2016. A. Higher transmission pathogens. **B.** lower transmission pathogens. The difference between panels is the Y-axis range, to better display the data. Lines represent age-seroprevalence estimated by treatment group using locally weighted regression over individual seropositivity data (points), trimmed to ages 3-28 months to avoid edge effects. Points and error bars to the right of each panel show treatment-specific marginal average seroprevalence and 95% confidence intervals by study visit (1, 2, 3). Gray shading shows child age ranges of visits 1 and 3, with the primary outcome and full cohort measured at visit 3. Similar estimates summarized by the WASH intervention margin are in **Figure 3**. WASH=combined water, sanitation and handwashing intervention.

Supplementary Table 4. Enteric pathogen seroprevalence at the 14-month visit by individual treatment group and main-effect comparison

Pathogen	Intervention group	Seroprevalence n/N (%)	Main effect group	Seroprevalence n/N (%)	RD (95% CI)	p-value
**Viruses**						
***Norovirus* GI/GII**	Control	77.9 (250/321)	No WASH	78.9 (520/659)	Ref	—
	WASH	75.1 (274/365)	WASH	75 (533/711)	-3.9 (-9.0, 1.2)	0.59
	Nutrition	79.9 (270/338)	No Nutrition	76.4 (524/686)	Ref	—
	WASH + Nutrition	74.9 (259/346)	Nutrition	77.3 (529/684)	0.9 (-4.2, 5.9)	0.89
**Bacteria**						
**LT-ETEC (LTB)**	Control	93.5 (300/321)	No WASH	92.3 (608/659)	Ref	—
	WASH	89.3 (326/365)	WASH	90.3 (642/711)	-2.0 (-5.4, 1.4)	0.62
	Nutrition	91.1 (308/338)	No Nutrition	91.3 (626/686)	Ref	—
	WASH + Nutrition	91.3 (316/346)	Nutrition	91.2 (624/684)	-0.1 (-3.5, 3.3)	0.97
***Campylobacter* (p18,p39)**	Control	97.5 (313/321)	No WASH	96.8 (638/659)	Ref	—
	WASH	97.8 (357/365)	WASH	97 (690/711)	0.2 (-1.7, 2.1)	0.97
	Nutrition	96.2 (325/338)	No Nutrition	97.7 (670/686)	Ref	—
	WASH + Nutrition	96.2 (333/346)	Nutrition	96.2 (658/684)	-1.5 (-3.4, 0.5)	0.69
***Shigella*/EIEC (IpaB)**	Control	21.5 (69/321)	No WASH	22.6 (149/659)	Ref	—
	WASH	24.1 (88/365)	WASH	24.6 (175/711)	2.0 (-3.2, 7.3)	0.66
	Nutrition	23.7 (80/338)	No Nutrition	22.9 (157/686)	Ref	—
	WASH + Nutrition	25.1 (87/346)	Nutrition	24.4 (167/684)	1.6 (-3.7, 6.8)	0.86
***S. enterica typhi* (HlyE)**	Control	7.5 (24/321)	No WASH	7 (46/659)	Ref	—
	WASH	6.8 (25/365)	WASH	7.2 (51/711)	0.2 (-2.6, 3.0)	0.97
	Nutrition	6.5 (22/338)	No Nutrition	7.1 (49/686)	Ref	—
	WASH + Nutrition	7.5 (26/346)	Nutrition	7 (48/684)	-0.1 (-2.9, 2.7)	0.97
S. enterica* (LPS B,D)	Control	9.7 (31/321)	No WASH	6.4 (42/659)	Ref	—
	WASH	4.7 (17/365)	WASH	4.9 (35/711)	-5.0 (8.4, -1.6)	0.05
	Nutrition	3.3 (11/338)	No Nutrition	7 (48/686)	Ref	—
	WASH + Nutrition	5.2 (18/346)	Nutrition	4.2 (29/684)	-6.4 (-9.8, -3.0)	0.00
***V. cholerae* (OSP-Inaba/Ogawa)**	Control	1.9 (6/321)	No WASH	1.8 (12/659)	Ref	—
	WASH	0.5 (2/365)	WASH	1.3 (9/711)	-0.5 (-1.9, 0.8)	0.66
	Nutrition	1.8 (6/338)	No Nutrition	1.2 (8/686)	Ref	—
	WASH + Nutrition	2 (7/346)	Nutrition	1.9 (13/684)	0.7 (-0.6, 2.1)	0.69

**Protozoa**						
***Cryptosporidium* (Cp17,Cp23)**	Control	53 (170/321)	No WASH	53.1 (350/659)	Ref	—
	WASH	58.6 (214/365)	WASH	57.1 (406/711)	4.0 (-2.2, 10.1)	0.62
	Nutrition	53.3 (180/338)	No Nutrition	56 (384/686)	Ref	—
	WASH + Nutrition	55.5 (192/346)	Nutrition	54.4 (372/684)	-1.5 (-3.4, 0.5)	0.86
***Giardia* (VSP-5)**	Control	10.3 (33/321)	No WASH	9.3 (61/659)	Ref	—
	WASH	11 (40/365)	WASH	10 (71/711)	0.7 (-2.5, 3.9)	0.91
	Nutrition	8.3 (28/338)	No Nutrition	10.6 (73/686)	Ref	—
	WASH + Nutrition	9 (31/346)	Nutrition	8.6 (59/684)	-2.0 (-5.2, 1.2)	0.69
***E. histolytica* (LecA)**	Control	1.6 (5/321)	No WASH	2.1 (14/659)	Ref	—
	WASH	1.4 (5/365)	WASH	1.5 (11/711)	-0.6 (-2.0, 0.9)	0.66
	Nutrition	2.7 (9/338)	No Nutrition	1.5 (10/686)	Ref	—
	WASH + Nutrition	1.7 (6/346)	Nutrition	2.2 (15/684)	0.7 (-0.7, 2.2)	0.69
**Helminths**						
***T. solium* (T24,Es33)**	Control	2.5 (8/321)	No WASH	2 (13/659)	Ref	—
	WASH	1.9 (7/365)	WASH	2 (14/711)	0.0 (-1.5, 1.5)	0.99
	Nutrition	1.5 (5/338)	No Nutrition	2.2 (15/686)	Ref	—
	WASH + Nutrition	2 (7/346)	Nutrition	1.8 (12/684)	-0.4 (-1.9, 1.1)	0.86
S. stercoralis* (NIE)	Control	0 (0/321)	No WASH	0.2 (1/659)	Ref	—
	WASH	0.5 (2/365)	WASH	0.3 (2/711)	0.1 (-0.3, 0.6)	0.99
	Nutrition	0.3 (1/338)	No Nutrition	0.3 (2/686)	Ref	—
	WASH + Nutrition	0 (0/346)	Nutrition	0.1 (1/684)	0.3 (-0.3, 0.9)	0.69

Note:
n/N (%) shows observed seropositive counts and percentages.
Risk differences are marginal main-effect estimates reported in percentage points.
*Conditional means and effects are reported for pathogens with evidence of additive interaction.
P values are two-sided permutation p-values from the primary main-effect models.

Pathogen	Nutrition	No Nutrition	Diff (95% CI)	p-value	FDR p-value
Norovirus GI/GII	77.4%	76.5%	0.9 (-4.2, 5.9)	0.74	0.89
LT-ETEC (LTB)	91.2%	91.3%	-0.1 (-3.5, 3.3)	0.97	0.97
Campylobacter (p18,p39)	96.2%	97.7%	-1.5 (-3.4, 0.5)	0.14	0.69
Shigella/EIEC (IpaB)	24.4%	22.8%	1.6 (-3.7, 6.8)	0.56	0.86
S. enterica typhi (HlyE)	7.0%	7.1%	-0.1 (-2.9, 2.7)	0.93	0.97
S. enterica (LPS B,D) *	3.3%	9.7%	-6.4 (-9.8, -3.0)	<0.01	<0.01
V. cholerae (OSP-Inaba/Ogawa)	1.9%	1.2%	0.7 (-0.6, 2.1)	0.29	0.69
Cryptosporidium (Cp17,Cp23)	54.4%	55.8%	-1.5 (-7.7, 4.7)	0.64	0.86
Giardia (VSP-5)	8.6%	10.6%	-2.0 (-5.2, 1.2)	0.23	0.69
E. histolytica (LecA)	2.2%	1.5%	0.7 (-0.7, 2.2)	0.34	0.69
T. solium (T24,Es33)	1.8%	2.2%	-0.4 (-1.9, 1.1)	0.57	0.86

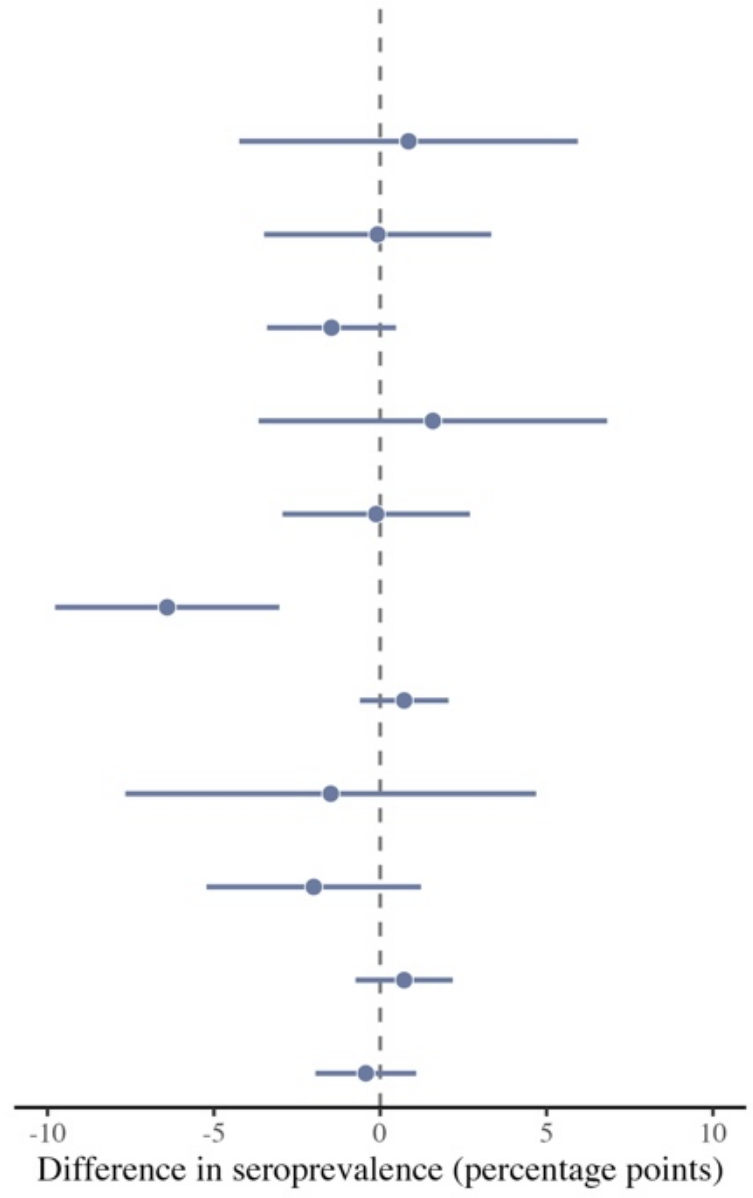

Supplementary Figure 4. Nutrition intervention effects on enteric pathogen seroprevalence at median age 14 months. Effects estimated as marginal means and treatment effects for clusters that received Nutrition (Nutrition, WASH + Nutrition) compared with clusters that did not (WASH, Control)**.** * Conditional means and effects comparing Nutrition vs Control are reported in cases of interaction between WASH and Nutrition interventions (P< 0.1) for *S. enterica* LPS Groups B and D. Two-sided permutation p-values were estimated by permuting treatment assignments (10,006 replicates). P-values were adjusted for multiple comparisons using the Benjamini-Hochberg procedure across the 12 comparisons (5% false discovery rate). Excluded results for *S. stercoralis* (NIE) because seroprevalence was low (<1%).

WASH=combined water, sanitation and handwashing; Diff=difference; 95% CI=95% confidence interval; FDR=False Discovery Rate.

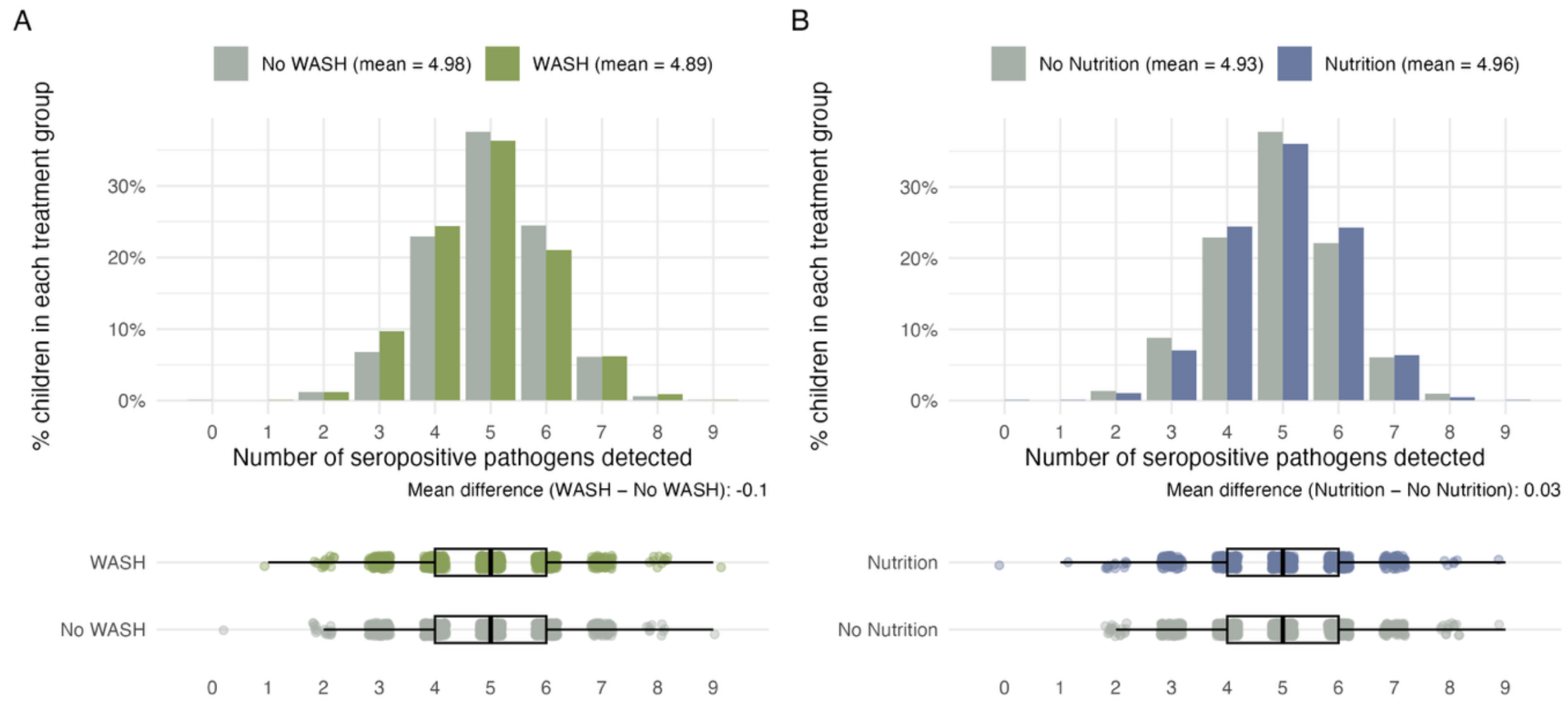

Supplementary Figure 5. Distribution of the number of seropositive enteric pathogens detected among children by WASH and Nutrition interventions at median age 24 months. WASH=combined water, sanitation and handwashing.
